# Gas-to-Particle Partitioning of Products from Ozonolysis of Δ^3^-Carene and the Effect of Temperature and Relative Humidity

**DOI:** 10.1021/acs.jpca.3c07316

**Published:** 2024-01-31

**Authors:** Linjie Li, Ditte Thomsen, Cheng Wu, Michael Priestley, Emil Mark Iversen, Jane Tygesen Sko̷nager, Yuanyuan Luo, Mikael Ehn, Pontus Roldin, Henrik B. Pedersen, Merete Bilde, Marianne Glasius, Mattias Hallquist

**Affiliations:** † Department of Chemistry and Molecular Biology, University of Gothenburg, Gothenburg 41296, Sweden; ‡ Department of Chemistry, Aarhus University, Aarhus 8000, Denmark; § Institute for Atmospheric and Earth System Research/Physics, University of Helsinki, Helsinki 00014, Finland; ∥ Department of Physics, Lund University, Lund 22100, Sweden; ⊥ IVL Swedish Environmental Institute, Malmö21119, Sweden; # Department of Physics and Astronomy, Aarhus University, Aarhus 8000, Denmark

## Abstract

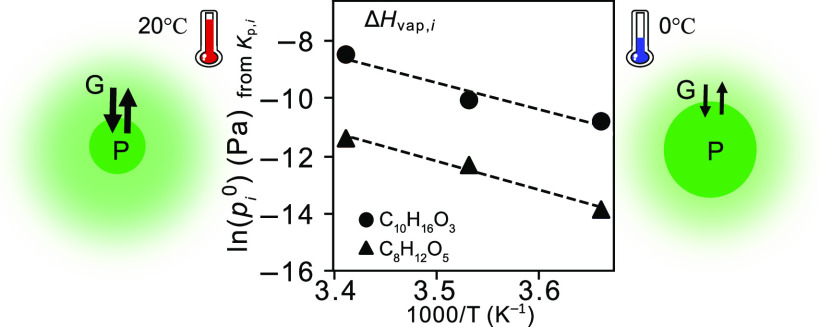

Formation of oxidized products from Δ^3^-carene (C_10_H_16_) ozonolysis and their gas-to-particle partitioning at three temperatures (0, 10, and 20 °C) under dry conditions (<2% RH) and also at 10 °C under humid (78% RH) conditions were studied using a time-of-flight chemical ionization mass spectrometer (ToF-CIMS) combined with a filter inlet for gases and aerosols (FIGAERO). The Δ^3^-carene ozonolysis products detected by the FIGAERO-ToF-CIMS were dominated by semivolatile organic compounds (SVOCs). The main effect of increasing temperature or RH on the product distribution was an increase in fragmentation of monomer compounds (from C_10_ to C_7_ compounds), potentially via alkoxy scission losing a C_3_ group. The equilibrium partitioning coefficient estimated according to equilibrium partitioning theory shows that the measured SVOC products distribute more into the SOA phase as the temperature decreases from 20 to 10 and 0 °C and for most products as the RH increases from <2 to 78%. The temperature dependency of the saturation vapor pressure (above an assumed liquid state), derived from the partitioning method, also allows for a direct way to obtain enthalpy of vaporization for the detected species without accessibility of authentic standards of the pure substances. This method can provide physical properties, beneficial for, e.g., atmospheric modeling, of complex multifunctional oxidation products.

## 1 Introduction

Secondary organic aerosols (SOAs) are formed through the oxidation of volatile organic compounds (VOCs) producing lower volatile products that are subjected to gas-particle partitioning.^1^ The SOAs may adversely affect human health and play a complex role in aerosol climate effects.^2^ A large source of SOA is the production of low volatile products from the oxidation of monoterpenes (C_10_H_16_).^3^ Monoterpenes are an important class of biogenic VOCs,^4^ including α-pinene, β-pinene, limonene, and Δ^3^-carene. The oxidation of α-pinene, which is emitted in large quantities, has received much attention in chamber, model, and ambient studies.^5−9^ Recent studies also suggest high importance for the monoterpene Δ^3^-carene.^10−12^ The study of the particle phase of Δ^3^-carene ozonolysis indicates the efficient condensing of the *cis*-3-caric acid product on existing particles.^10^ The alkoxy bond scission of the cyclohexyl from OH and NO_3_ oxidation of Δ^3^-carene may lead to further oxidized organic products.^11,12^

Although the emission of Δ^3^-carene is often lower than α-pinene, the oxidation pathways of Δ^3^-carene (initiated by O_3_, OH, and NO_3_) have been reported to give a higher yield of SOA mass compared to the structurally similar α-pinene (see structures in Figure S1).^10−14^ In addition, under specific conditions for species in the boreal forest, the actual emission of Δ^3^-carene can be the dominant monoterpene.^15,16^ The Δ^3^-carene oxidation therefore can be an important source of SOA and be comparable to aerosol formation from α-pinene oxidation at some scenarios.^14−17^ To further elucidate the role of Δ^3^-carene for ambient SOA formation, more studies on the effects of temperature and relative humidity (RH) would be required.

The gas-to-particle partitioning of oxidation products of monoterpenes is a key factor for SOA formation and its descriptions in atmospheric models.^18,19^ The particle fraction of the total concentration of a compound *i* (*F*_p,*i*_) is a direct parameter to describe partitioning, but it relies on the existing organic aerosol mass (*M*_org_).^20,21^ According to Raoult’s law described by Pankow,^22^ the equilibrium partitioning coefficient of a compound *i* (*K*_p,*i*_) is frequently applied to characterize equilibrium phase partitioning between the gas and aerosol phases of the compound *i*, and it remains constant across a certain range of *M*_org_ concentrations. In addition, the inverse of *K*_p,*i*_ of each compound links to its saturation concentration (*C*_i_*) and equilibrium saturation vapor pressure (*p*_*i*_^0^). Previous studies indicate that the conversion from the gas phase to particle phase of organic compounds is sensitive to RH, sulfate ions (SO_4_^2–^), and organic aerosol mass.^7,20,23−25^ However, the partitioning of oxidation products from ozonolysis of Δ^3^-carene under different temperatures and RH is still unknown. The enthalpies of vaporization and sublimation of individual oxidation products can be extracted from the temperature-dependent equilibrium saturation vapor pressure given that the phase state of the condensed phase is known.^26^ The enthalpy of change during the phase transition (Δ*H*_trs_) is a key thermodynamic property, describing the energy needed for the phase transition, which is used in the development of partitioning models.^20,24^

In this study, we investigated the effects of temperature and RH on gas-particle partitioning of products formed in the Δ^3^-carene ozonolysis. Both the gas- and particle-phase concentrations of individual compounds from Δ^3^-carene ozonolysis were measured using a filter inlet for gases and aerosols (FIGAERO) coupled to a high-resolution time-of-flight chemical ionization mass spectrometer (HR-ToF-CIMS) enabling detailed insight into the gas-to-particle partitioning of oxidation products.^27,28^ In total, we investigate three dark and dry (RH < 2%) ozonolysis experiments at temperatures of 0, 10, and 20 °C, respectively, and one high RH (78%) ozonolysis experiment at a temperature of 10 °C of Δ^3^-carene in the Aarhus University Research on Aerosol (AURA) atmospheric simulation chamber.^29^ Simultaneous gas and particle measurements enable the application of equilibrium partitioning theory^22^ to derive partitioning coefficients of detected products. Subsequently, the experiments at different temperatures offer an innovative way to derive enthalpy of vaporization for detected species based on the assumption that the particles exist in a liquid state.

## 2 Materials and Methods

### 2.1 Chamber and Sampling

A series of dark ozonolysis experiments of Δ^3^-carene were conducted during the Aarhus Chamber Campaign on Evaporation, Partitioning, and Terpene Oxidation (ACCEPTO). To scrutinize the effect on the formation of oxidized products and their gas-to-particle partitioning, three dry experiments at three temperatures (0, 10, and 20 °C) and one high RH (78% and 10 °C) experiment run in the batch mode were selected (Table 1). An overview of ACCEPTO and the detailed AURA atmospheric simulation chamber setup and the experiment conditions are described by Thomsen et al.^30^ In short, AURA is a ∼5 m^3^ temperature-controlled Teflon chamber.^29^ Ozone (∼180 ppb, ozone generator model 610, Jelight Company, Inc.) and Δ^3^-carene (∼10 ppb, Sigma-Aldrich, >98.5%) were injected into a zero-air-filled AURA chamber, and the resulting evolution of products in gas and particle phases was observed for a total experimental time of 4 h. The ozone concentration was monitored by a UV photometric analyzer (O342 Module, Environnement S.A.). The temperature and RH were measured by a probe (HC2-C04, Rotronic AG) in the middle of the chamber. Aerosol mass was measured by a high-resolution time-of-flight aerosol mass spectrometer (AMS, Aerodyne Research, Inc.) and a scanning mobility particle sizer (SMPS, model 3082, Impactor 071CM, TSI, Inc.). A number of instruments were connected to the AURA chamber. In this work, the focus was to use the data obtained using HR-ToF-CIMS combined with a FIGAERO inlet (FIGAERO-ToF-CIMS, Aerodyne Research, Inc.). In addition, the concentration of *M*_org_ was derived from measurements using the SMPS and the AMS.

**Table 1:** Experimental Conditions

ID	exp. date	Δ^3^-carene (ppb)	ozone (ppb)	temp. (°C)	RH (%)
dry, 0 °C	220202	10 ± 5	159 ± 15	0.1 ± 0.1	1.6 ± 1.6
dry, 10 °C	220204	10 ± 5	171 ± 15	10.1 ± 0.1	0.4 ± 0.8
dry, 20 °C	220205	10 ± 5	181 ± 15	20.2 ± 0.1	0 ± 0
humid, 10 °C	220131	10 ± 5	174 ± 15	10.2 ± 0.1	77.8 ± 1.9

### 2.2 FIGAERO-ToF-CIMS

The HR-ToF-CIMS detected the products in a negative ionization mode. The reagent ion, iodide (I^–^), was provided from the ionization of methyl iodide (CH_3_I) by a polonium-210 radioactive source and forms a cluster with the analyte molecule (MI^–^) in the ion–molecule reactor (IMR). The FIGAERO inlet was operated in a 56 min cycle and measured 5 cycles during each experiment. In each cycle, the gas phase was measured for 10 min with a 2 L per minute (LPM) flow from the AURA chamber. The sampling line was an 80 cm-long perfluoroalkoxy (PFA) Teflon tube with a 4 mm inner diameter. For gas-phase backgrounds, pure nitrogen (N_2_) purged the inlet for 1 min with 2 LPM flow before and after gas-phase measurements. During the gas-phase measurement (in total 10 min), the particles were simultaneously collected on a PALL PTFE membrane filter at a flow rate of 3 LPM. Particle collection was followed by a 44 min thermal desorption, which was performed by heated N_2_ over the filter from room temperature to 200 °C with a 15 min temperature ramp, then followed by holding the temperature constant at 200 °C for 20 min and a 9 min cooling down to room temperature. The desorption profile reveals a maximum desorption temperature (*T*_max_), which has a nearly linear correlation to the natural logarithm of *p*_*i*_^0^.^31,32^ The HR-ToF-CIMS detected compounds with an average spectral resolution of 3000 (*m*/Δ*m*), and the data analysis was processed using Tofware v3.2.5. Mass calibrants, such as I^–^ (126.904 *m*/*z*), H_2_OI^–^(144.915 *m*/*z*), C_2_H_4_O_2_I^–^ (acetic acid, 186.926 *m*/*z*), I_3_^–^ (380.713 *m*/*z*), C_5_HF_9_O_2_I^–^ (perfluoropentanoic acid, 390.888 *m*/*z*), and C_7_HF_13_O_2_I^–^ (perfluoroheptanoic acid, 490.881 *m*/*z*), were used to convert time-of-flight to *m*/*z*. The formulas of all detected products were derived from high-resolution multipeak fitting results of the individual peak. The integrated signals (ions/s) of each molecular formula were extracted from the raw data. One may note that for each identified molecular formula, there might be several isomers.

### 2.3 Gas-to-Particle Partitioning Coefficient

The measured signal in the HR-ToF-CIMS was normalized and background-corrected to enable comparison of trends and relative contributions at the various conditions. Shortly, extracted ion signals were normalized by the intensity of the reagent I^–^ and multiplied by 10^6^. Gas-phase signals were corrected by the chamber background. The particle-phase signals were corrected using the filter blank conducted during the same experiment. The sensitivity of the iodide adduct HR-ToF-CIMS is usually constrained by the collision limit of reagent ions and analytes, the ion–molecule reaction time, and the ion transmission efficiency.^33,34^ However, due to the resistance caused by the filter, the N_2_ flow is different as reported by Lopez-Hilfiker et al.;^34^ thus, the sensitivity may vary. The IMR pressure is controlled by a pressure controller. The conversion of the gas-phase signal to gas concentration and how particle-phase signal was corrected for the sampling volume are presented in the SI together with a discussion on the effect of the inlet flow. The gas-phase signal and the volume-corrected particle-phase signal were used to calculate the particle concentration to gas concentration ratio. After evaporation, the molecules from the particle phase were ionized in the IMR and detected by the CIMS in the same way as the gas phase. One can assume the same sensitivity in calculating the particle concentration to gas concentration ratio, i.e., the sensitivity for each compound is not needed in deriving the ratio.

According to Raoult’s law and absorptive gas-to-particle partitioning theory, the partitioning coefficient, *K*_p*,i*_, of individual species can be calculated according to eq 1.^22^
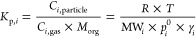
1Here, *C*_*i*,gas_ and *C*_*i*,particle_ are the concentrations of compound *i* in the gas phase and the particle phase. The ratios of measured signals are equivalent to the ratio of *C*_*i*,particle_/*C*_*i*,gas_ in eq 1. MW_*i*_ is the molecular weight of each species, *p*_*i*_^0^ is the saturation vapor pressure of each species, γ_*i*_ is the activity coefficient assumed to be 1, *R* is the universal gas constant, and *T* is the temperature in Kelvin (K). Conditions and timescales for reaching equilibrium of organic compounds depend on the particle-phase state.^35,36^ For a typical chamber study, the equilibrium timescale for gas-particle partitioning is on the order of minutes for semivolatile organic compounds (SVOCs) with a C* of 10 μg m^–3^, and the equilibrium time of more volatile oxidation products is shorter.^35,36^ The term C* is typically used to express volatility and can be calculated based on *p*_*i*_^0^. Consequently, the first particle sample (experiment time from 0 to 10 min after injection of Δ^3^-carene) was not considered in order to reduce the impact of initial nucleation and particle growth on the equilibrium assumption. Rather, the subsequent particle samples and corresponding gas-phase measurements were used for the calculation of *K*_p*,i*_. In addition to obtaining a *K*_p*,i*_ for each *C*_*i*,particle_/*C*_*i*,gas_ ratio, one can derive *K*_p*,i*_ from the slope of all derived *C*_*i*,particle_/*C*_*i*,gas_ ratios as a function of *M*_org_ as described in eq 1. Here, the coefficient of determination (*R*^2^) assuming a linear relationship between *C*_*i*,particle_/*C*_*i*,gas_ and *M*_org_, according to eq 1, could be used to rank the quality of the derived *K*_p*,i*_, i.e., the slope of *C*_*i*,particle_/*C*_*i*,gas_ versus *M*_org_. Furthermore, the linear regression fitting was used to describe the uncertainties of *K*_p*,i*_. Uncertainties of the measurement, i.e., ion signals measured by HR-ToF-CIMS and *M*_org_, are represented at the 95% confidence level of the averages of the collected data for selected time periods. The variability of *M*_org_ is within ±1%, and the variability of ion signals measured by HR-ToF-CIMS is within ±5%. The detection limit of each species detected by HR-ToF-CIMS is calculated as 3 times of the standard deviation (std) of the backgrounds.

### 2.4 Estimation of Vapor Pressure and Enthalpy from the Partitioning Coefficient

Using the derived partitioning coefficient *K*_p*,i*_, one may calculate the saturation concentration *C*_i_* or the corresponding equilibrium saturation vapor pressure *p*_*i*_^0^ (Pa) of compound *i* from eqs 2 or 3, respectively.^20,31^
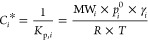
2
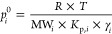
3

Conventionally, organic aerosols in the atmosphere are assumed to be in the liquid state, and the organic compounds forming part of the aerosol can be considered to be in equilibrium with the gas phase.^26,37^ One may note that if, for any reason, the produced particles are not in the liquid phase or being amorphous, one would not expect a linear relationship between *C*_*i*,particle_/*C*_*i*,gas_ and *M*_org_. Here, the derived *p*_*i*_^0^ is assumed to be the vapor pressure of compound *i* in the subcooled liquid-phase state. Additionally, the partitioning is temperature-dependent.^20,24−26^ The temperature dependence of *p*_*i*_^0^ can be described using the Clausius–Clapeyron equation (eq 4) where *A* is a constant.^26^
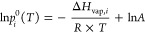
4Thus, the slope of the logarithm of *p*_*i*_^0^ versus reciprocal temperature is related to the enthalpy of vaporization (Δ*H*_vap,*i*_). The uncertainties of Δ*H*_vap,*i*_ are derived from the fitting of the ln *p*_*i*_^0^ versus 1/*T* relationship. Here, Δ*H*_vap,*i*_ is assumed to be temperature-independent over the relatively small temperature range studied.^24^

For a single compound, the temperature at the maximum evaporation rate, *T*_max_, during the particle-phase desorption from the FIGAERO filter is related to its vapor pressure.^31,32^ In recent studies, a relationship between *T*_max_ and *p*_*i*_^0^ has been reported for carboxylic acids and polyethylene glycol standards.^28,32,38^ However, the explicit relationship can be influenced by the FIGAERO–CIMS setup, temperature ramp, and mass loading. If *T*_max_ is derived for a specific *m*/*z*, one may also consider the possibilities for multiple isomers to contribute to the signal. In this study, the *T*_max_ values were extracted using a Gaussian fitting of multipeaks by the Gothenburg University Fitting for Thermograms (GUFIT).^32^ Most detected compounds have only one prominent maximum and will provide a unique peak *T*_max_. The fitted *T*_max_ of monomers is generally lower than 120 °C.^39^ For *T*_max_ higher than 120 °C, one may expect a significant contribution from thermal decomposition of compounds with higher *m*/*z*. This can often result in observations of several maxima of *T*_max_ from the GUFIT.^7^

The effective C* of individual compounds can also be calculated based on molecular formula parametrizations. One updated parametrization published by Mohr et al.^40^ (eq 5) is used here for comparison, where *n*_0_ = 25, *b*_C_ = 0.475, *b*_O_ = 0.2, *b*_CO_ = 0.9, *b*_N_ = 2.5, and *n*_C_, *n*_O_, and *n*_N_ are the number of carbon, oxygen, and nitrogen atoms in the compound, respectively. A parametrization described by Peräkylä et al.^41^ for α-pinene ozonolysis products was also used (eq 6), where *n*_H_ is the number of hydrogens. The temperature dependence of C* (270 < *T* < 330 K) follows eqs 7 and 8 based on the method by Epstein et al.^24^
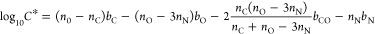
5
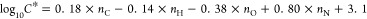
6
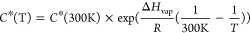
7
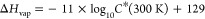
8

## 3 Results and Discussion

### 3.1 Formation and Evolution of Oxidation Products

**Figure 1:**
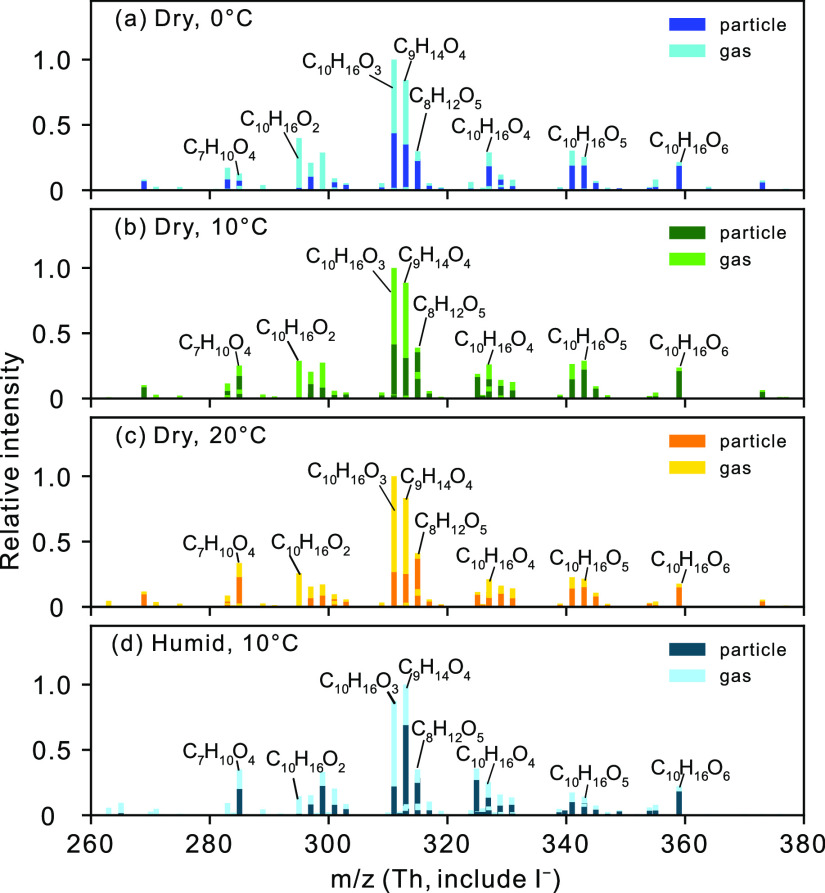
Monomer region mass spectra of gas and particle phases from Δ^3^-carene + O_3_ experiments at around 2 h experiment time. (a) Mass spectra from the dry, 0 °C experiment, (b) mass spectra from the dry, 10 °C experiment, (c) mass spectra from the dry, 20 °C experiment, and (d) mass spectra from the humid, 10 °C experiment. Oxidation products were detected as adducts with the iodide reagent ion (I^–^), but the iodide is omitted from the given product formulas. All mass spectra are normalized to the highest peak in a given experiment. Specific data on the indicated ions are found in Table S1.

**Figure 2:**
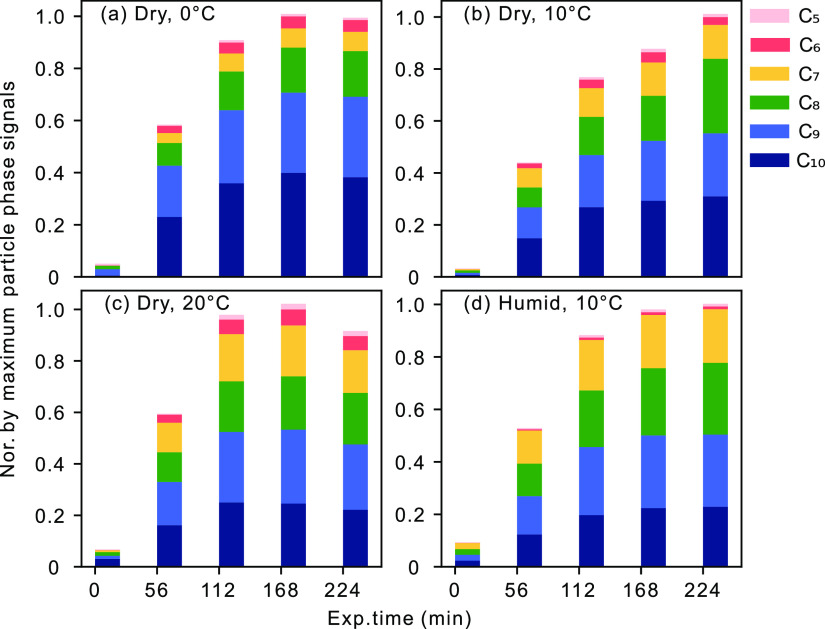
(a–d) Distribution of monomer products in the particle phase over the experiment time (exp. time) (0–10, 56–66, 112–122, 168–178, and 224–234 min) according to carbon numbers. All distributions are normalized to the maximal particle-phase signals in a given experiment.

In the Δ^3^-carene experiments, 240 product ions were identified using the FIGAERO-ToF-CIMS. Monomers, defined as products with 5 to 10 carbon atoms, were the dominant products (Figure 1), and their signals contributed to more than 88% of the total signals of gas and particle phases of all detected products at all temperatures and humidities. The relative distributions of the monomers had similar patterns for all three dry experiments, i.e., dominated by C_10_H_16_O_*n*_ (being *n* = 2–6) and some smaller compounds (C_7_–C_9_). The two products with the highest signal intensities, corresponding to the molecular formulas C_10_H_16_O_3_ and C_9_H_14_O_4_, are likely caronic acid and caric acid, respectively, which have been reported as dominant early-stage products of Δ^3^-carene ozonolysis.^10^ The more oxidized compounds, C_10_H_16_O_4–6_, contributed more (*F*_p,*i*_ in range of 0.32–0.89) to the particle phase than the less oxidized compounds, i.e., C_10_H_16_O_2–3_ (*F*_p,*i*_ in range of 0.03–0.44) (Figure 1 and Table S1). Other dominant compounds such as C_7_H_10_O_4_ and C_9_H_14_O_4_ showed presence (Figure 1 and Table S1) in both the gas and particle phases, while C_8_H_12_O_5_ was mainly detected in the particle phase. The molecular formula C_8_H_12_O_5_ could be an analogue compound to 2-hydroxyterpenylic acid that is a product formed in the oxidation of α-pinene.^42^ This compound is often observed to be abundant in ambient particles.^43^ The amount of all detected monomer products in the particle phase increased rapidly during the first 2 h of the experiment followed by a more stable period where their concentrations exhibited only small changes (Figure 2). This is in good agreement with Thomsen et al.^44^ who found a similar increase in the level of carboxylic acids in the particle phase from ozonolysis of Δ^3^-carene.

The temperature effect on the product distribution can be seen for the gas and particle phases in Figure 1 and tabulated in Table S1. To provide more detailed information, Figure 2 shows the effect of temperature on the monomer categories with carbon numbers 5–10. Here, the fraction of the C_10_ category decreases from around 39 to 25% when the temperature increases from 0 to 20 °C, while the fraction of C_7_ rises from 7 to 19%. Clearly, fragmentation of the parent carbon skeleton increases with higher temperatures. Meanwhile, the contribution of C_10_H_16_O_4–6_, slightly more oxidized compounds compared to C_10_H_16_O_2–3_, does not show a temperature dependency (Figure 1 and Table S1). This does not align with previous studies that noted that more oxygenated compounds were favored at higher temperatures.^45−47^ This could be because only compounds with up to 6 oxygen atoms were identified in this study. Alkoxyl radical scission can explain the production of the smaller compounds (C_5_–C_9_). Except for C_7_ compounds, the fraction of the total phase concentration of the smaller identified compounds did not exhibit a dependence on temperature in the AURA chamber (Figure 2). This has been observed in previous Δ^3^-carene reacted with OH and NO_3_ studies where abundant C_7_ resulted from losing a C_3_ group of the cyclopropyl ring-opened alkoxy radical.^11,12^

High RH has also been found to affect aerosol yields and composition.^23,48,49^ Here, we observe some products, e.g., C_9_H_14_O_4_ and C_7_H_10_O_4_, to increase (Figure 1b,d and Table S1) and the fraction of C_10_ compounds to decrease at high RH in the particle phase (Figure 2b,d). The intensity of the product C_9_H_14_O_4_ is the highest under the humidified condition (Figure 1d and Figure S2). Water can influence the product distribution in several different ways. Compared to the dry experiments, water molecules may react with intermediates from Δ^3^-carene ozonolysis, which will change the radical chemistry in the gas phase.^23,49^ Also at high RH, water affects the partitioning behavior by increasing condensation to the humidified particles.^23,49^

### 3.2 Partitioning Coefficients

**Figure 3:**
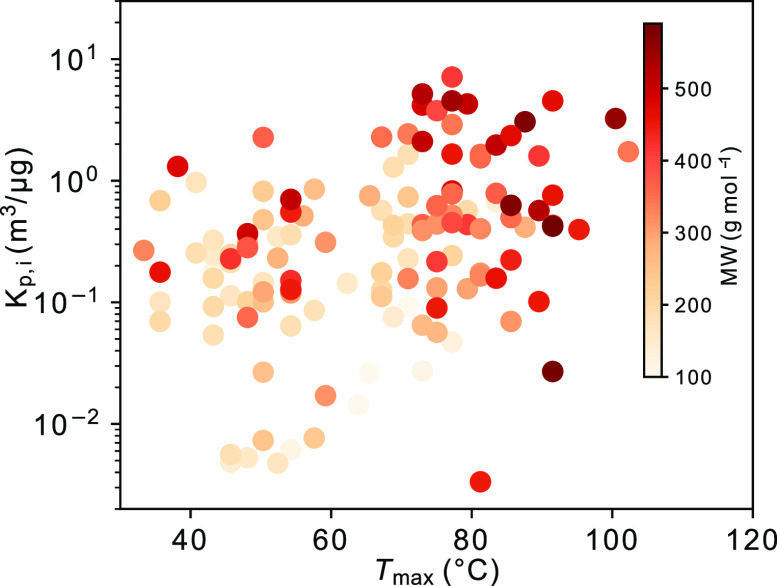
*K*_p*,i*_ derived for the dry, 20 °C experiment at around 2 h versus *T*_max_ of oxidation compounds labeled with their molecular weight (MW).

**Figure 4:**
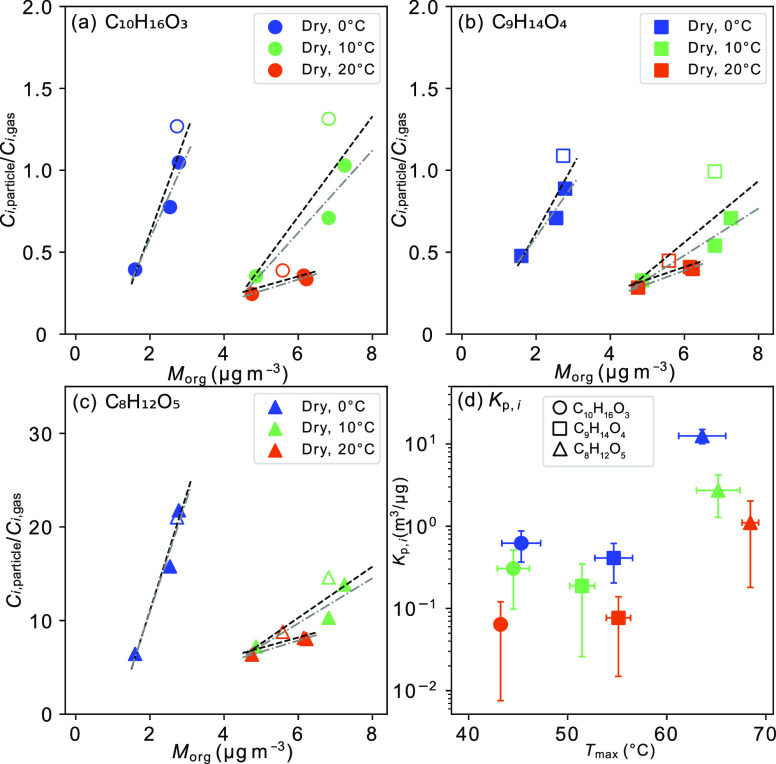
*C*_*i*,particle_/*C*_*i*,gas_ ratio versus organic aerosol mass (*M*_org_) for (a) product C_10_H_16_O_3_, (b) product C_9_H_14_O_4_, (c) product C_8_H_12_O_5_, and (d) the three products’ *K*_p,*i*_ against *T*_max_. According to eq 1, the slope of the linear fit is equal to the partitioning coefficients, *K*_p,*i*_. The black dashed line is the linear fit from exp. times of 56–66, 112–122, 168–178, and 224–234 min, and the gray dash-dotted line is the linear fit from exp. times of 56–66, 112–122, and 168–178 min. The open points are the last samples from the exp. time of 224–234 min. The three products’ *K*_p,*i*_ in (d) were extracted from the black dashed line fitting in the dry 0, 10, and 20 °C experiments (blue: 0 °C, green: 10 °C, and orange: 20 °C). The *K*_p,*i*_ error bar is the statistical uncertainty at the 95% confidence level of the linear regression fitting. The *T*_max_ error bar is the corresponding standard deviation of *T*_max_ values from exp. times of 56–66, 112–122, 168–178, and 224–234 min.

**Figure 5:**
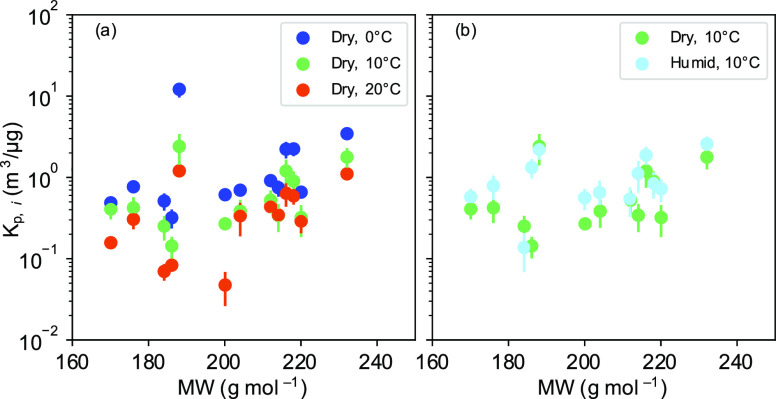
*K*_p*,i*_ values of the 13 compounds in Table 2. (a) Dry, 0 °C experiment, dry, 10 °C experiment, and dry, 20 °C experiment and (b) humid, 10 °C experiment. *K*_p,*i*_ values are derived from *C*_*i*,particle_/*C*_*i*,gas_ ratios versus *M*_org_ during the experiment times (56–66, 112–122, and 168–178 min). The *K*_p*,i*_ error bar is the uncertainty of *K*_p*,i*_.

The *C*_*i*,particle_/*C*_*i*,gas_ ratios of all species, which were detected in amounts above their detection limits in both the gas and particle phases, were calculated. This was the case for 160 compounds. The *C*_*i*,particle_/*C*_*i*,gas_ ratio for an individual compound is sensitive to temperature and RH (Figure S3) but also on *M*_org_. The produced *M*_org_ varies between experiments and changes during an experiment as reactions proceed. Assuming that equilibrium has been established, one may compensate for the dependency on *M*_org_ by calculating the partitioning coefficient, *K*_p*,i*_, using eq 1. This can be done for each individual point or from the slope of *C*_*i*,particle_/*C*_*i*,gas_ versus *M*_org_ within each experiment. Figure 3 shows the values of *K*_p,*i*_ for products measured in the dry, 20 °C experiment at 2 h as derived from eq 1. This experiment is close to ambient temperature, and the SOA mass concentration peaked at around 2h and then decreased due to the wall loss (Figure S4). A previous study reported that *T*_max_ increases with higher *K*_p*,i*_ and MW.^7^ For the products from Δ^3^-carene ozonolysis, there is a tendency between *T*_max_ and both *K*_p,*i*_ and MW, but the correlation is relatively weak (Figure 3). The *T*_max_ method is more suitable for determining *p*_*i*_^0^ of thermally stable, low-volatility compounds with a high MW (≥190 g mol^–1^),^6,50^ while *K*_p,*i*_ values are frequently studied for SVOC compounds.^7,26,51^ To further investigate the methodology for determining *K*_p,*i*_ for selected compounds, the slope method, based on more data points from each experiment, was used. A comparison between the slope method and the point method can be found in Figure S5. The *K*_p,*i*_ values obtained through the point method have the same trend as those derived from the slope method but with a bias toward lower values (Figure S5). As an example of using the slope method, the *C*_*i*,particle_/*C*_*i*,gas_ versus *M*_org_ for three selected products from Δ^3^-carene ozonolysis, representing both low and high *C*_*i*,particle_/*C*_*i*,gas_ ratios, C_10_H_16_O_3_, C_9_H_14_O_4_, and C_8_H_12_O_5_, is shown in Figure 4. The slope of their *C*_*i*,particle_/*C*_*i*,gas_ ratios versus *M*_org_ represents the *K*_p*,i*_ (Figure 4a–c). The uncertainty of the fitted slope providing *K*_p,*i*_ is still quite high even for the selected 13 products with *R*^2^ (≥0.44) between *C*_*i*,particle_/*C*_*i*,gas_ and *M*_org_ (Figure S6), and one may note the curved shape in Figure 4a–c. This is probably due to dilution and wall loss of gases and particles in the chamber that increases over time, i.e., *M*_org_ is lower toward the end of the experiment. A lower *M*_org_ should give a lower *C*_*i*,particle_/*C*_*i*,gas_, but if the time for equilibrium is slow, one may observe a high *C*_*i*,particle_/*C*_*i*,gas_ value at that instant. Furthermore, if the fitting excludes the last sample, which is likely most affected by the effects of dilution and the wall loss (exp. time from 224 to 234 min) (Figures S4 and S6), the fit to a linear relationship improves significantly, and the uncertainties on resulting *K*_p*,i*_ become quite small (Figure 5). A list of *K*_p,*i*_ of the 13 products with high *R*^2^ (≥0.8) for a linear relationship between *C*_*i*,particle_/*C*_*i*,gas_ and *M*_org_ together with their molecular formulas under the different experiments can be found in Table 2. The *K*_p,*i*_ values of caronic acid (C_10_H_16_O_3_) and caric acid (C_9_H_14_O_4_) for all experiments are derived in the ranges of 0.07–0.51 and 0.08–1.32 m^3^/μg, respectively (Table 2). They are at the similar levels of predicted *K*_p,*i*_ values for pinonic acid (0.034 m^3^/μg) and pinic acid (0.31 m^3^/μg) at 25 °C formed from α-pinene.^52^ The *K*_p,*i*_ of C_8_H_12_O_5_ (an analogue compound to 2-hydroxyterpenylic acid) is up to 12 m^3^/μg at 0 °C.

**Table 2:** Calculated *K*_p*,i*_ for the 13 Compounds with the Strong *R*^2^ (≥0.8) to a Linear Fit of *C*_*i*,particle_/*C*_*i*,gas_ vs *M*_org_ (from Exp. Times of 56–66, 112–122, and 168–178 min) and Average of *T*_max_ Extracted from Exp. Times of 56–66, 112–122, and 168–178 min

	*T*_**max**_**(°C)** (mean ± SD)				**partitioning coefficient (***K*_**p,***i*_**) (m**^**3**^**/μg)(***K*_**p,***i*_**± uncertainty)**				
									**Δ***H*_**vap,***i*_
**formula**	dry, 0 °C	dry, 10 °C	dry, 20 °C	humid, 10 °C	dry, 0 °C	dry, 10 °C	dry, 20 °C	humid, 10 °C	(kJ mol^–1^)
C_9_H_14_O_3_	47.0 ± 1.5	48.4 ± 0.5	45.1 ± 3.1	49.2 ± 1.4	0.49 ± 0.09	0.41 ± 0.10	0.16 ± 0.01	0.58 ± 0.15	40 ± 16
C_6_H_8_O_6_	42.0 ± 0.9	47.1 ± 2.5	48.7 ± 1.1	45.3 ± 2.0	0.77 ± 0.07	0.42 ± 0.15	0.31 ± 0.08	0.79 ± 0.26	33 ± 6
C_10_H_16_O_3_	45.3 ± 2.0	44.5 ± 1.6	43.2 ± 1.2	44.8 ± 3.0	0.51 ± 0.12	0.25 ± 0.08	0.07 ± 0.02	0.14 ± 0.07	68 ± 12
C_9_H_14_O_4_	54.6 ± 1.9	51.5 ± 1.3	55.1 ± 1.2	47.6 ± 1.4	0.32 ± 0.08	0.14 ± 0.04	0.08 ± 0.01	1.32 ± 0.35	47 ± 4
C_8_H_12_O_5_	63.6 ± 2.4	65.2 ± 2.2	68.4 ± 0.8	52.2 ± 3.5	12.13 ± 2.52	2.41 ± 1.01	1.20 ± 0.12	2.22 ± 0.57	80 ± 16
C_10_H_16_O_4_	57.0 ± 3.3	50.0 ± 17.4	49.6 ± 18.0	51.8 ± 4.8	0.61 ± 0.03	0.27 ± 0.01	0.05 ± 0.02	0.56 ± 0.16	87 ± 19
C_8_H_12_O_6_	47.8 ± 2.4	54.0 ± 1.0	68.0 ± 1.2	49.0 ± 1.2	0.70 ± 0.12	0.38 ± 0.14	0.34 ± 0.15	0.65 ± 0.26	27 ± 8
C_6_H_12_O_8_	54.7 ± 9.8	50.7 ± 3.7	48.7 ± 1.1	44.7 ± 13.7	0.91 ± 0.15	0.52 ± 0.17	0.44 ± 0.06	0.54 ± 0.21	27 ± 7
C_10_H_14_O_5_	48.4 ± 2.3	46.9 ± 3.0	49.0 ± 3.0	49.6 ± 1.7	0.75 ± 0.17	0.34 ± 0.13	0.34 ± 0.05	1.12 ± 0.47	28 ± 15
C_10_H_16_O_5_	48.4 ± 2.2	48.4 ± 0.6	48.8 ± 1.3	51.9 ± 2.9	2.22 ± 0.51	1.20 ± 0.45	0.64 ± 0.20	1.88 ± 0.51	44 ± 1
C_9_H_14_O_6_	55.1 ± 3.4	58.0 ± 6.2	72.4 ± 6.1	51.5 ± 2.7	2.24 ± 0.42	0.90 ± 0.29	0.59 ± 0.13	0.85 ± 0.30	47 ± 9
C_8_H_12_O_7_	53.6 ± 2.8	53.8 ± 2.2	68.0 ± 1.2	51.4 ± 1.1	0.66 ± 0.01	0.32 ± 0.14	0.29 ± 0.08	0.72 ± 0.23	30 ± 11
C_10_H_16_O_6_	42.8 ± 0.4	45.2 ± 1.6	49.2 ± 1.3	50.2 ± 1.2	3.45 ± 0.50	1.78 ± 0.52	1.11 ± 0.14	2.59 ± 0.59	40 ± 3

### 3.3 Temperature and RH Dependency on the Partitioning, *K*_p,*i*_

The *K*_p*,i*_ values of the three carboxylic acids show a negative dependence on temperature in the chamber (Figure 4d). The *K*_p*,i*_ values of the 13 compounds in Table 2 with a strong correlation (*R*^2^ ≥ 0.8) between *C*_*i*,particle_/*C*_*i*,gas_ and *M*_org_ also present clear temperature dependence (Table 2 and Figure 5). The *K*_p*,i*_ values exhibit an increase with each 10 °C decrease in temperature that can be attributed to the change in equilibrium vapor pressures. The *K*_p*,i*_ values of most of the 13 compounds increase under humidified conditions probably because of enhanced particle uptake at higher RH.^23^ Oxidation products are also most likely hydrophilic consisting of alcohol (R–OH), hydroperoxides, and carboxylic (R–C(=O)OH) groups that facilitate the condensation into the SOA phase.^23^

Based on measured *T*_max_ values of C_9_H_14_O_4_ (51–55 °C) and C_10_H_16_O_3_ (43–45 °C) and the literature,^7,28^ the *K*_p,*i*_ of caric acid (C_9_H_14_O_4_) is expected to be higher than the *K*_p,*i*_ of caronic acid (C_10_H_16_O_3_). However, the *K*_p,*i*_ of C_9_H_14_O_4_ is slightly lower than the *K*_p,*i*_ of C_10_H_16_O_3_ in the 0 and 10 °C experiments. The measurement of products from the analogue α-pinene ozonolysis suggests that the I-CIMS-detected formula C_9_H_14_O_4_ in the gas phase may include a peracid, which may lead to lower *C*_*i*,particle_/*C*_*i*,gas_ ratios and *K*_p,*i*_ of C_9_H_14_O_4_.^53^

### 3.4 Vapor Pressure and Parameterization

**Figure 6:**
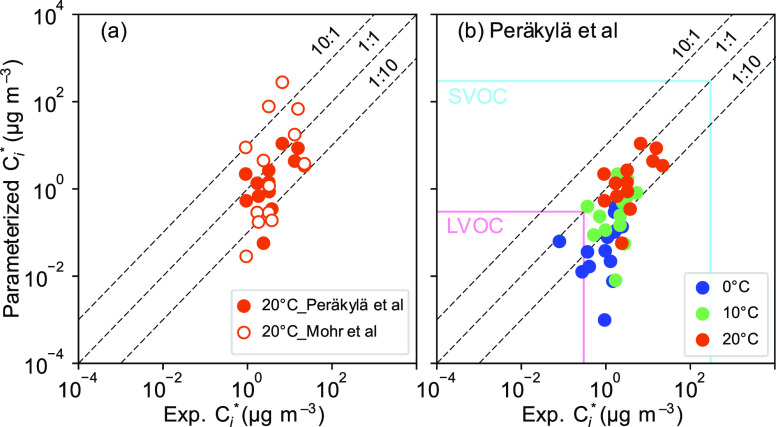
Molecular formula-parametrized *C*_i_* versus experimental *C*_i_* derived from *K*_p*,i*_. (a) Molecular formula-parametrized *C*_i_* based on Mohr et al.^40^ and Peräkylä et al.^41^ and experimental *C*_i_* of the dry, 20 °C experiment; (b) molecular formula-parametrized *C*_i_* based on Peräkylä et al.^41^ and experimental *C*_i_* of the dry 0, 10, and 20 °C experiments.

The inverse of *K*_p*,i*_ provides *C*_i_* (eq 2), and with some assumption (ideal mixture), one can also derive *p*_*i*_^0^ from *K*_p*,i*_ (eq 3). *C*_i_* has shown to be a useful quantity to describe the volatility of SOA formation and may together with knowledge on the oxidation state provide a so-called two-dimensional volatility basis set (2-D-VBS).^20,54^ Organic molecules in the atmosphere can be categorized into VOCs with C* > 3 × 10^6^ μg m^–3^, intermediate-volatility organic compounds (IVOCs) (300 < C* < 3 × 10^6^ μg m^–3^), SVOCs (0.3 < C* < 300 μg m^–3^), low-volatility organic compounds (LVOCs) (3 × 10^–5^ < C* < 0.3 μg m^–3^), and extremely low-volatility organic compounds (ELVOCs) with C* < 3 × 10^–5^ μg m^–3^ based on their C*.^20,54^ Many published parametrizations also reported the effective C* taking into account the influence of nonideal thermodynamic mixing for oxygenated compounds in both ambient and chamber studies.^40,41,55^ The *C*_i_* values of the compounds in Table 2 were calculated from two of these molecular formula parametrizations (eqs 5^40^ and 6(41)). In Figure 6a, we compared the parametrized *C*_i_* and the experimental *C*_i_* from our observation of gas-to-particle partitioning of the 13 compounds in the experiment at 20 °C, which is close to the temperatures at which the parametrizations were developed. The *C*_i_* values derived in this study match better with parametrized *C*_i_* values based on Peräkylä et al.^41^ than based on Mohr et al.^40^ This can likely be understood by the experiment-based parametrization method of Peräkylä et al.^41^ being more applicable to chamber studies than the field study-based parametrization method of Mohr et al.^40^ Thus, we compared the parametrized *C*_i_* values at 0 and 10 °C based on Peräkylä et al.,^41^eqs 7 and 8,^24^ and *C*_i_* from the dry, 0 °C and dry, 10 °C experiments in Figure 6b. The experimentally derived values of *C*_i_* lie within the parametrized range of values of *C*_i_*. These compounds are SVOCs at the conditions in the dry, 10 °C and dry, 20 °C experiments, while the volatility of some compounds shifts toward LVOC at the colder temperature (Figure 6b). The derived *K*_p*,i*_ is related to the vapor pressures (eq 1). Very few vapor pressure measurements exist on products from Δ^3^-carene oxidation.^26,56^ However, the derived vapor pressure for C_10_H_16_O_3_, assumed to be caronic acid, is comparable to measured values (derived from measured evaporation rates of dried particles) of the analogue product pinonic acid produced from α-pinene oxidation, i.e., 1.90 × 10^–4^ Pa for caronic acid (Table S2) compared to 7 × 10^–5^,^57^ 0.42 × 10^–5^,^58^ and 1.29 × 10^–4^ Pa^59^ for pinonic acid. One may note that commonly used estimation methods deviate considerably and give much higher vapor pressures for pinonic acid, e.g., 1.4 × 10^–2^ to 3.9 × 10^–3^ Pa.^6^ This is also valid when comparing the parametrized values for carene products shown in Figure 6 with values directly calculated using the SIMPOL^60^ or EVAPORATION^61^ methods, e.g., for caronic acid, *p*_*i*_^0^ (300 K) is 1.18 × 10^–1^ (EVAPORATION) and 1.68 × 10^–2^ Pa (SIMPOL) vs 3.2 × 10^–3^ (eq 5) and 4.52 × 10^–4^ Pa (eq 6). One may note that the assumption on the activity coefficient of one when deriving *p*_i_^0^ from *K*_p*,i*_ has some uncertainties.^62^ This clearly illustrates the need for a future extension on the work on vapor pressures for the complex multifunctional compounds found in the oxidation of monoterpenes.

**Figure 7:**
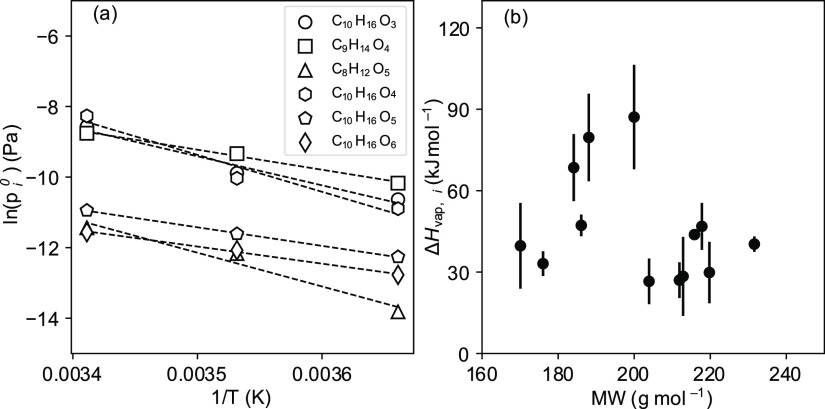
Slope of the logarithm of *p*_*i*_^0^ versus reciprocal temperature. According to eq 4, the slope of the linear fit is equal to Δ*H*_vap,*i*_/*R* if the particles are in the liquid state. (a) ln(*p*_*i*_^0^) extracted from eq 3 versus the 1/*T* in Kelvin of products C_10_H_16_O_3_, C_9_H_14_O_4_, C_8_H_12_O_5_, C_10_H_16_O_4_, C_10_H_16_O_5_, and C_10_H_16_O_6_; (b) Δ*H*_vap,*i*_ of products in Table 2 versus their molecular weight. The Δ*H*_vap,*i*_ error bar is at the 95% confidence level of the linear regression fitting.

The temperature dependence of *C*_i_* can be described using the Clausius–Clapeyron relationship, if Δ*H*_vap_ is known.^20,24^ Δ*H*_vap_ signifies the energy needed for the phase transition from liquid to gas.^26^ It can be extracted from the temperature-dependent equilibrium vapor pressure (eq 4).^24,26^ Δ*H*_vap,*i*_ in this study is extracted from a linear least-squares fit of ln *p*_*i*_^0^ versus the inverse of temperature (Figure 7a). The Δ*H*_vap_ values calculated based on *K*_p*,i*_ from both the point method and slope method are also consistent (Figure S7). The Δ*H*_vap,*i*_ values for the selected SVOC compounds produced in the ozonolysis of Δ^3^-carene as derived from the temperature-dependent *K*_p*,i*_ fall in the range of 27 to 87 kJ mol^–1^ (Figure 7b and Table 2). For caric acid (C_10_H_16_O_4_), the Δ*H*_vap_ was determined to be 47 ± 4 kJ mol^–1^. There exist no other measurements of Δ*H*_vap_ for these compounds, but the values are of the same magnitude and broadly consistent at the lower end with those of polyfunctional acids.^26^

For some α-pinene oxidation products, previous studies have reported enthalpies of sublimation (Δ*H*_sub_) or vaporization (Δ*H*_vap_) derived using evaporation of the pure authentic compounds.^57,58^ The Δ*H* for the transition from the condensed particle phase to gas phase of pinic acid has been measured in two separate studies to be 83 ± 5 and 109 ± 21 kJ mol^–1^, respectively.^57,58^ As discussed by Bilde et al.,^26^ it can be difficult to determine the physical phase state of the particles if not explicitly probed. It is commonly assumed that organic particles in air are subcooled liquid aerosol instead of crystalline particles;^37^ thus, Δ*H*_vap_ could be more relevant than Δ*H*_sub_ for atmospheric models. Δ*H*_vap_ is typically lower than Δ*H*_sub_ at a given temperature, and the difference is the enthalpy of fusion (Δ*H*_fus_). This may explain the difference between the derived Δ*H*_vap_ of caric acid in this work and Δ*H*_sub_ of pinic acid.^57,58^ More work is needed to understand these differences, but as such, the FIGAERO–CIMS method to derive Δ*H*_vap_ in temperature-dependent chamber studies can be a very important tool enabling characterization of the huge range of multifunctional compounds that lack authentic standards.

### 3.5 Atmospheric Implication

The FIGAERO–CIMS allows for simultaneous characterization of the gas-phase and particle-phase composition. The measurement of gas-to-particle partitioning provides the crucial parameters, *K*_p,*i*_ and Δ*H*_vap,*i*_ of the oxidation compounds, for modeling SOA formation.^20,22,24,57^ The observed *K*_p,*i*_ values of C_10_H_16_O_3_ were found to be 0.51 ± 0.12, 0.25 ± 0.08, and 0.07 ± 0.02 m^3^/μg for the dry, 0 °C, dry, 10 °C, and dry, 20 °C experiments, respectively. This is in line with *K*_p,*i*_ of C_10_H_16_O_3_ (0.32 m^3^/μg) measured in a field study conducted at an ambient temperature of approximately 4 °C.^7^ The observed Δ*H*_vap_ of C_10_H_16_O_3_ (68 ± 12 kJ mol^–1^), which is likely caronic acid, is close to the reported Δ*H*_sub_ of the corresponding α-pinene oxidation analogue pinonic acid (90 ± 7 kJ mol^–1^).^58^ While broadly consistent, the difference between them could be Δ*H*_fus_.^63^ Our study demonstrates the ability of using a new gas-to-particle partitioning method for determining *K*_p,*i*_ and Δ*H*_vap,*i*_ for SVOCs in chamber studies using the iodide-adduct-FIGAERO.

Nonetheless, this study has some limitations. Primarily, the wall loss of semivolatile organic compounds (SVOCs) in the Teflon chamber has been reported,^64,65^ which can affect the accuracy of partitioning measurement. However, this study did not assess the specific wall loss for each individual compound. The heat-induced evaporation of the particle phase using the FIGAERO inlet also comes with certain limitations. For example, the mass loading on the filter may affect the derived *T*_max_ from the thermograms, and the thermogram may also include information from multiple isomers and thus different volatilities.^9,28,31,66^ The variations in sensitivities of different isomers may also affect the measurement of partitioning. However, the requirements used to derive partitioning coefficients and the corresponding heat of evaporation rely on a consistent partitioning behavior as a function of *M*_org_ and temperature. If a specific formula consists of several dominant isomers, they must exhibit similar partitioning behavior as a function of *M*_org_ and temperature to provide linear relationships. Still, these potential limitations will impact the precision of the *K*_p,*i*_. The calculation of Δ*H*_vap,*i*_ based on derived *K*_p,*i*_ also encounters a notable degree of uncertainty in Δ*H*_vap,*i*_ estimation.

However, in contrast to frequently employed experimental techniques for Δ*H* determination (such as Knudsen effusion mass spectrometry, flow tube tandem differential mobility analyzer, volatility tandem differential mobility analyzer, and thermal desorption particle beam mass spectrometry), the FIGAERO method offers the potential to measure Δ*H*_trs_ associated with the transition from the condensed SOA phase to the gas phase of a multitude of compounds. Further research in both field and chamber studies of SOA, the FIGAERO method could explore the Δ*H* values of more compounds over a wider temperature range.

## 4 Conclusions

In this study, the SOA formation from dark Δ^3^-carene ozonolysis at different temperatures (0, 10, and 20 °C) and RH (dry and 78%) were investigated using a FIGAERO-ToF-CIMS. The particles from Δ^3^-carene ozonolysis form, grow, and stabilize in the AURA atmospheric simulation chamber over a 4 h reaction time. The oxidized compounds detected in the highest amounts are in the monomer region dominated by C_10_H_16_O_2–6_ and smaller compounds (e.g., C_9_H_14_O_4_, C_8_H_12_O_5_, and C_7_H_10_O_4_) in both gas and particle phases. The C_10_H_16_O_3_ product maximizes during the dry experiments, while the C_9_H_14_O_4_ product maximizes during the high RH experiment, which are likely caronic acid and caric acid, respectively. The fragmentation of C_10_ becomes more prominent at higher temperatures and high RH, which leads to an increase in the C_7_ fraction. The *K*_p,*i*_ and *C*_i_* of the measured oxidation products were estimated from slope *C*_*i*,particle_/*C*_*i*,gas_ ratios against *M*_org_ concentrations. *K*_p*,i*_ values of compounds in Table 2 increase as temperatures decrease from 20 to 10 °C and from 10 to 0 °C, and *K*_p*,i*_ values of most compounds also increase at high RH. More experiments with different RH levels can be conducted to investigate the RH impact on Δ^3^-carene ozonolysis in further research.

The temperature-dependent *C*_i_* and *p*_*i*_^0^ derived from *K*_p*,i*_ show a temperature dependence where the volatility decreases at the colder temperature. Assuming a liquid particle state, the Δ*H*_vap_ values of selected compounds calculated using temperature-dependent *p*_*i*_^0^ had a range from 27 to 87 kJ mol^–1^. Although the initial measurement of Δ*H*_vap_ was limited to 13 compounds and exhibited a high level of uncertainty in this study, the FIGAERO method demonstrates the potential to derive Δ*H*_vap_ of more complex compounds, for which there are no authentic standards.

## Data Availability

Data are available on request from the corresponding author: Mattias Hallquist (hallq@chem.gu.se). The data will also be available through the ATMO-ACCESS platform (https://data.eurochamp.org/). The data used in this manuscript are listed in the tables, figures, and Supporting Information.

## Supporting Information Available

Materials and methods and supporting discussions; details of FIGAERO-ToF-CIMS signals and the comparison of partitioning coefficients (*K*_p,*i*_); supporting figures of molecular structures of α-pinene and Δ^3^-carene, mass spectra, particle concentration to gas concentration ratios, SOA mass concentration, and comparison of *K*_p,*i*_ and Δ*H*_vap_ (Figures S1–S7); supporting tables of the indicated monomer compounds and the equilibrium saturation vapor pressure *p*_*i*_^0^ (Tables S1 and S2) (PDF)
