# Supplementary material for: Gas-to-Particle Partitioning of Products from Ozonolysis of Δ3-Carene and the Effect of Temperature and Relative Humidity
Source: J Phys Chem A. 2024 Jan 31;128(5):918–28. doi: 10.1021/acs.jpca.3c07316 (PMC10860141; doi:10.1021/acs.jpca.3c07316)
Supplement: Supplementary file 1 — jp3c07316_si_001.pdf [file jp3c07316_si_001.pdf]

Supporting Information for

Gas-to-Particle Partitioning of Products from Ozonolysis of Δ^3-carene and the Effect of Temperature and Relative Humidity

Linjie Li[1], Ditte Thomsen[2], Cheng Wu[1], Michael Priestley[1,a], Emil Mark Iversen[2], Jane Tygesen Skønager[2], Yuanyuan Luo[3], Mikael Ehn[3], Pontus Roldin[4,5], Henrik B. Pedersen[6], Merete Bilde[2], Marianne Glasius[2], and Mattias Hallquist[1,*]

[1] Department of Chemistry and Molecular Biology, University of Gothenburg, Gothenburg, Sweden

[2] Department of Chemistry, Aarhus University, Aarhus, Denmark

[3] Institute for Atmospheric and Earth System Research/Physics, University of Helsinki, Helsinki, Finland

[4] Department of Physics, Lund University, Lund, 22100, Sweden

[5] IVL Swedish Environmental Institute, Malmö 21119, Sweden

[6] Department of Physics and Astronomy, Aarhus University, Aarhus, Denmark

[a] Now at: IVL Swedish Environmental Institute, Gothenburg 41133, Sweden

*Corresponding Author: Mattias Hallquist (hallq@chem.gu.se)

Content of this file

Text 1: Ion signals to mass concentration of FIGAERO-HR-ToF-CIMS.

Text 2: Method comparison for deriving partitioning coefficients ($K_{p,i}$).

Figure S1: Molecular structures of α-pinene and Δ^3-carene.

Figure S2: Monomer region mass spectra of gas and particle phases from humid, 10 °C-2 experiment.

Figure S3: $C_{i,\mathrm{particle}}$ to $C_{i,\mathrm{gas}}$ ratios of dominant products from the dry, 0 °C, dry, 10 °C, dry, 20 °C, and humid, 10 °C experiments.

Figure S4: The SOA mass concentration measured by SMPS over experiment time during the dry, 20°C experiment.

Figure S5: $K_{p,i}$ derived from each $C_{i,\mathrm{particle}}$ to $C_{i,\mathrm{gas}}$ ratio against M_{org} point versus $K_{p,i}$ derived from the slope of $C_{i,\mathrm{particle}}$ to $C_{i,\mathrm{gas}}$ ratio versus M_{org}.

Figure S6: $K_{p,i}$ of the 13 compounds derived from the experiment times (56–66 min, 112–122 min, 168–178 min, 224–234 min).

Figure S7: ΔH_{vap} derived from the point method versus ΔH_{vap} derived from the slope method.

Table S1: Selected monomer compounds from Δ^3-carene + O_3 experiments at around 2 hours experiment time.

Table S2: The equilibrium saturation vapor pressure p_i^0 (Pa) of compound *i* derived from the corresponding partitioning coefficient $K_{p,i}$.

Materials and Methods

Ion signals to mass concentration of FIGAERO-ToF-CIMS

To convert the ion signals to mass concentration in the gas phase, the extracted ion counts per second (cps) were normalized by reagent ion I^{-}, multiplied by 10^6 and divided by a sensitivity factor 20 counts s^{-1} ppt^{-1}.[1-4] The sensitivity factor differs based on the setup of the instrument and the chemical species being analyzed. For the particle phase, the integrated signal during the desoption was used to calculate the concentration of respective species. Here the sampling volume (duration and flow) was taken into account to properly compare to the gas phase measurement done during the same time interval. Our primary focus in this study was on partitioning; therefore, here we discuss the variations in sensitivity between gas-phase and particle-phase measurements.

First, the setup of the instrument parameters was the same in both gas phase and particle phase measurements. The iodide flow (F_{iodide}) is always 2 liters per minute (LMP) for both phases. The sample (gas-phase) flow (F_{gas}) is 2 LMP. When the FIGAERO inlet changes to the particle phase (desorption mode), the sample (particle phase) flow ($F_{particle}$) was set to 2 LPM, however, due to the resistance induced by the filter, the real flow going to the IMR was 1.6 LPM. The IMR pressure was compensated by the pressure controller by adjusting the IMR pumping outflow during the campaign. The difference in the flows into the IMR changes the residence time (t) of the analyte molecules in the IMR and the dilution of sampling flow ($F_{sampling}$) due to the iodide flow. At a certain IMR pressure, the residence time can be represented by the physical volume of the IMR (V_{IMR}) and the total volumetric flow (V_{IMR} /(F_{iodide} + $F_{sampling}$)). The reduced flow gave 1.11 longer residence time for the particle phase measurement. The dilution factor can be represented by ($F_{sampling}$ /(F_{iodide} + $F_{sampling}$)). Here the reduced flow gave a reduction in dilution by a factor of 0.89 for the particle phase. Thus, the overall correction factor on the ratio between particle and gas phase measurement considering

both residence time and dilution will then be 0.99 (1.11 × 0.89). Thus, ion signals of the gas phase and particle phase after this correction can be directly compared and the ratio could be calculated.

Method comparison for Deriving Partitioning Coefficients ($K_{p,i}$)

The $K_{p,i}$ values of the 13 compounds derived from the point method and the slope method are illustrated in Figure S5. Note that there are four values for each compound from the point measurements while the slope method provides one value. To elucidate the effect on the temperature dependency (ΔH_{vap}) point data at around 2 hours in each experiment were used (Figure S7). Here no obvious bias or deviating trend could be observed and one may conclude that the two methods are providing consistent values. However, in the main manuscript, the slope method was preferred since it provides information using data from several measurement points.

Supplementary Figures

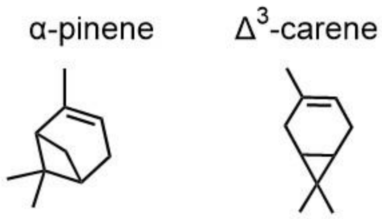

Figure S1. Structural information of α-pinene and Δ^3-carene. Molecular structures are depicted using ChemDraw version 22.2.0.

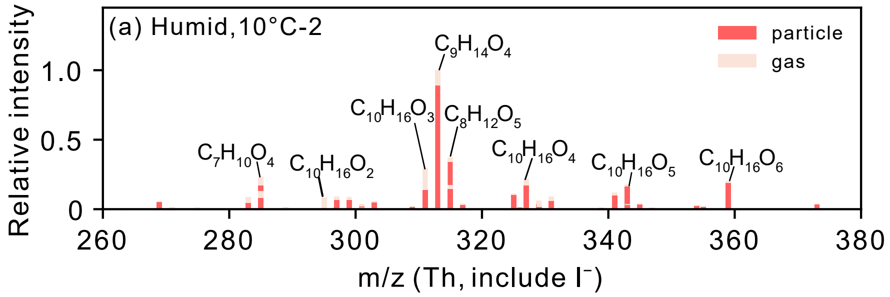

Figure S2. Monomer region mass spectra of gas and particle phases from the humid, 10 °C-2 experiment at around 2 hours exp. time. The humid, 10 °C-2 experiment is conducted with 20 ppb Δ^3-carene and 169 ppb O_3 injected into the chamber at high RH (76%) and 10 °C. The m/z value is the adduct product with (I^-). The mass spectra are normalized to the corresponding maximal compound.

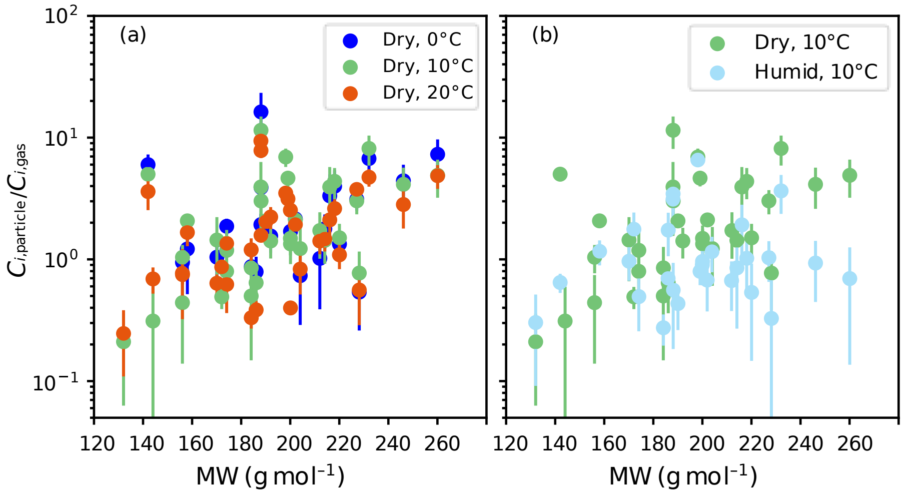

Figure S3. $C_{i,\mathrm{particle}}$ to $C_{i,\mathrm{gas}}$ ratios of the top 30 products detected by CIMS in both the gas and particle phases. (a) the dry, 0 °C experiment, the dry, 10 °C experiment, and the dry, 20 °C experiment, (b) the dry, 10 °C experiment and the humid, 10 °C experiment. The $C_{i,\mathrm{particle}}/C_{i,\mathrm{gas}}$ ratio here is the average of $C_{i,\mathrm{particle}}/C_{i,\mathrm{gas}}$ ratio values from experiment time 56–66 min, 112–122 min, 168–178 min, and 224–234 min during each experiment and the error bar is the corresponding standard deviation.

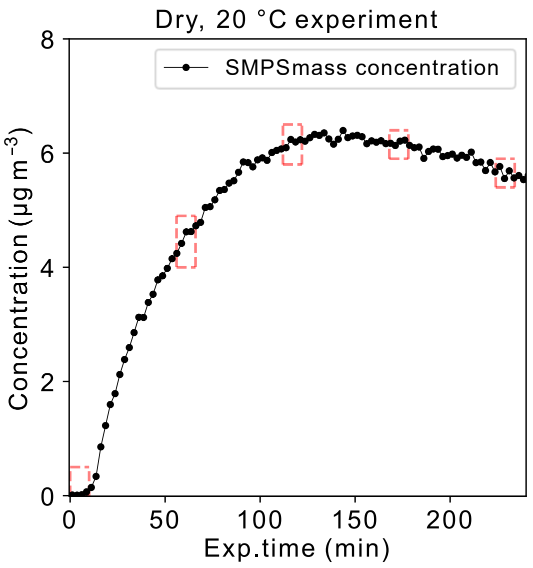

Figure S4. The SOA mass concentration measured by SMPS over experiment time during the dry, 20°C experiment. The time slots in the red frame correspond to the FIGAERO-CIMS sampling time 0–10 min, 56–66 min, 112–122 min, 168–178 min and 224–234 min.

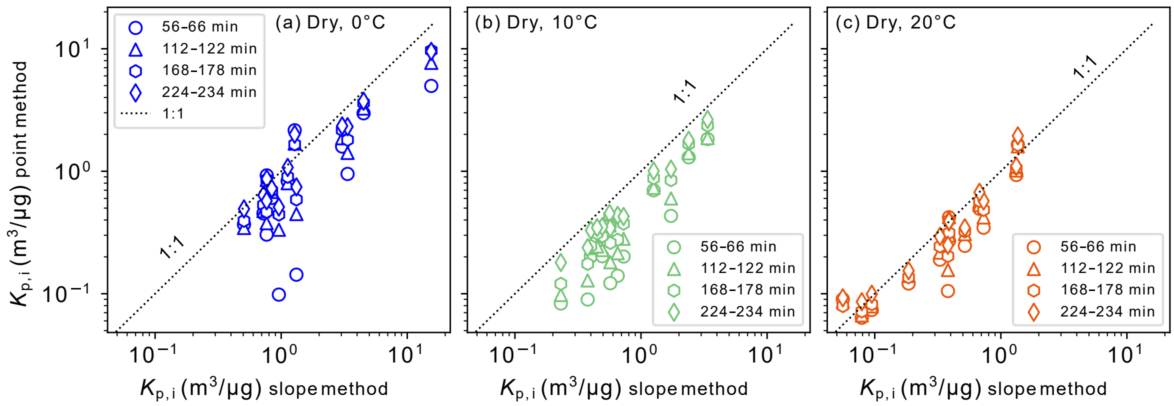

Figure S5: $K_{p,i}$ of the set of compounds in Table 2 dervied from each individual point from experiment time 56–66 min, 112–122 min, 168–178 min, and 224–234 min separately against the slope of $C_{i,\mathrm{particle}}/C_{i,\mathrm{gas}}$ versus M_{org} during experiment time (56–66 min, 112–122 min and 168–178 min).

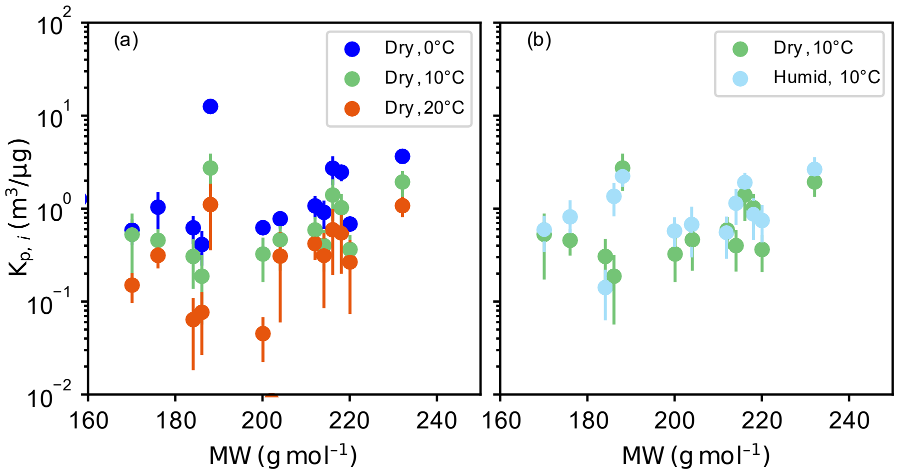

Figure S6. $K_{p,i}$ of the 13 compounds in Table 2. $K_{p,i}$ values are derived from $C_{i,\mathrm{particle}}/C_{i,\mathrm{gas}}$ ratios versus organic aerosol mass during experiment time (56–66 min, 112–122 min, 168–178 min, 224–234 min). The error bar is given at the 95% confidence level of the linear regression fitting.

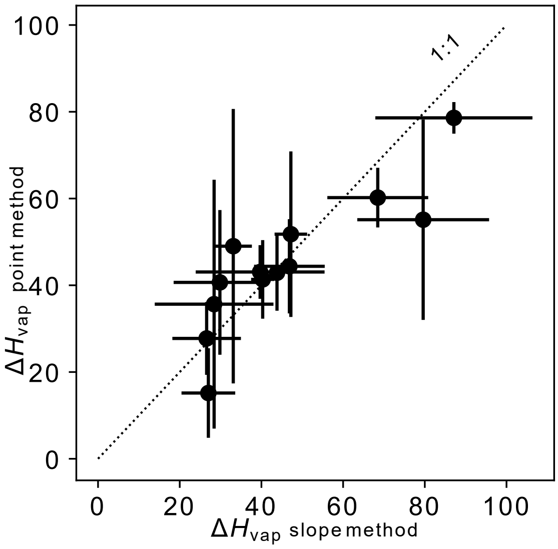

Figure S7: ΔH_{vap} of the 13 compounds in Table 2 derived from $K_{p,i}$ extracted from experiment time at around (112–122 min) versus ΔH_{vap} dervied from $K_{p,i}$ derived from the slope of particle to gas ratios versus M_{org} during experiment time (56–66 min, 112–122 min, 168–178 min). The error bar is given at the 95% confidence level of the linear regression fitting.

Supplementary Tables

Table S1. Selected monomer compounds from Δ^3-carene + O_3 experiments at around 2 hours experiment time, i.e. the marked formulas in Figure 1. Total ion signals are the sum of gas phase and particle phase signals. Ion signals are the extracted ion counts per second (cps) normalized by reagent ion I^-, multiplied by 10^6. $F_{p,i}$ is the particle fraction.

Compounds	MW (g mol^{-1})	Dry, 0 °		Dry, 10 °C		Dry, 20 °C		Humid, 10 °C	
		total ion signals	$F_{p,i}$	total ion signals	$F_{p,i}$	total ion signals	$F_{p,i}$	total ion signals	$F_{p,i}$
$C_7H_{10}O_4$	158.06	142	0.57	797	0.69	1295	0.68	400	0.58
$C_{10}H_{16}O_2$	168.12	442	0.05	910	0.04	995	0.03	165	0.08
$C_{10}H_{16}O_3$	184.11	1108	0.44	3157	0.41	3866	0.27	998	0.25
$C_9H_{14}O_4$	186.09	935	0.41	2797	0.35	3219	0.30	1143	0.69
$C_8H_{12}O_5$	188.07	264	0.94	1228	0.91	1590	0.90	400	0.82
$C_{10}H_{16}O_4$	200.11	320	0.64	823	0.57	818	0.32	290	0.53
$C_{10}H_{16}O_5$	216.10	282	0.74	914	0.77	834	0.70	101	0.73
$C_{10}H_{16}O_6$	232.10	238	0.87	749	0.89	686	0.85	254	0.82

Table S2 The equilibrium saturation vapor pressure p_i^0 (Pa) of compound *i* derived from the corresponding partitioning coefficient $K_{p,i}$, applying Equation 3 and assuming an activity coefficient of 1.

**Formula**	MW (g mol^{-1})	**Vapor pressure (p_i^0, Pa)** Dry, 0 °C	Dry, 10 °C	Dry, 20 °C	Humid, 10°C
$C_9H_{14}O_3$	170.10	2.7×10^{-5}	3.4×10^{-5}	9.1×10^{-5}	2.4×10^{-5}
$C_6H_8O_6$	176.03	1.7×10^{-5}	3.2×10^{-5}	4.5×10^{-5}	1.7×10^{-5}
$C_{10}H_{16}O_3$	184.11	2.4×10^{-5}	5.1×10^{-5}	1.9×10^{-4}	9.3×10^{-5}
$C_9H_{14}O_4$	186.09	3.8×10^{-5}	8.8×10^{-5}	1.6×10^{-4}	9.6×10^{-6}
$C_8H_{12}O_5$	188.07	1.0×10^{-6}	5.2×10^{-6}	1.1×10^{-5}	5.6×10^{-6}
$C_{10}H_{16}O_4$	200.11	1.8×10^{-5}	4.4×10^{-5}	2.6×10^{-4}	2.1×10^{-5}
$C_8H_{12}O_6$	204.06	1.6×10^{-5}	3.0×10^{-5}	3.5×10^{-5}	1.8×10^{-5}
$C_6H_{12}O_8$	212.05	1.2×10^{-5}	2.1×10^{-5}	2.6×10^{-5}	2.1×10^{-5}
$C_{10}H_{14}O_5$	214.09	1.4×10^{-5}	3.2×10^{-5}	3.3×10^{-5}	9.9×10^{-6}
$C_{10}H_{16}O_5$	216.10	4.7×10^{-6}	9.1×10^{-6}	1.8×10^{-5}	5.8×10^{-6}
$C_9H_{14}O_6$	218.08	4.6×10^{-6}	1.2×10^{-5}	1.9×10^{-5}	1.3×10^{-5}
$C_8H_{12}O_7$	220.06	1.6×10^{-5}	3.3×10^{-5}	3.8×10^{-5}	1.5×10^{-5}
$C_{10}H_{16}O_6$	232.10	2.8×10^{-6}	5.7×10^{-6}	9.5×10^{-6}	3.9×10^{-6}

Reference

(1) Mohr, C.; Lopez-Hilfiker, F. D.; Yli-Juuti, T.; Heitto, A.; Lutz, A.; Hallquist, M.; D'Ambro, E. L.; Rissanen, M. P.; Hao, L.; Schobesberger, S. Ambient observations of dimers from terpene oxidation in the gas phase: Implications for new particle formation and growth. *Geophysical Research Letters* **2017**, *44* (6), 2958-2966.

(2) Lutz, A.; Mohr, C.; Le Breton, M.; Lopez-Hilfiker, F. D.; Priestley, M.; Thornton, J. A.; Hallquist, M. Gas to Particle Partitioning of Organic Acids in the Boreal Atmosphere. *Acs Earth Space Chem* **2019**, *3* (7), 1279-1287. DOI: 10.1021/acsearthspacechem.9b00041.

(3) Lopez-Hilfiker, F. D.; Iyer, S.; Mohr, C.; Lee, B. H.; D'Ambro, E. L.; Kurtén, T.; Thornton, J. A. Constraining the sensitivity of iodide adduct chemical ionization mass spectrometry to multifunctional organic molecules using the collision limit and thermodynamic stability of iodide ion adducts. *Atmos. Meas. Tech.* **2016**, *9* (4), 1505-1512. DOI: 10.5194/amt-9-1505-2016.

(4) Lee, B. H.; Lopez-Hilfiker, F. D.; Mohr, C.; Kurtén, T.; Worsnop, D. R.; Thornton, J. A. An Iodide-Adduct High-Resolution Time-of-Flight Chemical-Ionization Mass Spectrometer: Application to Atmospheric Inorganic and Organic Compounds. *Environ Sci Technol* **2014**, *48* (11), 6309-6317. DOI: 10.1021/es500362a.
